# Timing of antihypertensive medication (bedtime *versus* morning) and cardiovascular risk: an updated systematic review and meta-analysis

**DOI:** 10.3389/fphar.2026.1758890

**Published:** 2026-03-30

**Authors:** Tuo Wang, Sijia Lai, Dayang Wang, Xian Wang

**Affiliations:** 1 Dongzhimen Hospital, Beijing University of Chinese Medicine, Beijing, China; 2 Geriatrics Department, Beijing Hospital of Traditional Chinese Medicine, Capital Medical University, Beijing, China; 3 Institute of Cardiovascular Diseases, Beijing University of Chinese Medicine, Beijing, China

**Keywords:** ABPM, bedtime, chronotherapy, hypertension, MACE, mortality, nocturnal

## Abstract

**Introduction:**

Previous studies on the optimal timing for antihypertensive medication administration have yielded inconsistent results. While earlier meta-analyses indicated that bedtime dosing significantly reduces myocardial infarction (MI) risk, more recent research has presented conflicting evidence. Therefore, we aimed to evaluate the impact of bedtime *versus* morning dosing of antihypertensive drugs on cardiovascular outcomes.

**Methods:**

We conducted a systematic search across PubMed, EMBASE, and Web of Science databases, covering studies published from inception to 16 July 2025, to identify relevant randomized controlled trials. We calculated risk ratios (RRs) for dichotomous outcomes along with the corresponding 95% confidence intervals (CIs). The study protocol was registered in PROSPERO (ID: CRD420251102968).

**Results:**

In total, 8 studies involving 64,235 patients were included in our analysis. Compared with morning dosing, bedtime dosing exhibited no significant differences in all-cause mortality (RR: 0.78; 95% CI: 0.59–1.04; *P* = 0.09), major adverse cardiovascular events (RR: 0.80; 95% CI: 0.61–1.05; *P* = 0.10) or cardiovascular death (RR: 0.59; 95% CI: 0.34–1.04; *P* = 0.07). However, bedtime dosing significantly decreased heart failure risk (RR: 0.67; 95% CI: 0.46–0.96; *P* = 0.03), lowered clinical systolic blood pressure (mean difference: −3.27 mmHg; 95% CI: −4.13 to −2.41; *P* < 0.00001), and improved non-dipper conversion (RR: 0.61; 95% CI: 0.47–0.77; *P* < 0.0001). Non-significant trends favoring bedtime dosing were noted for MI and coronary revascularization.

**Conclusion:**

For critical outcomes, including MACE, all-cause mortality and cardiovascular death, no statistically significant benefits associated with bedtime dosing were observed. The overall findings are inconsistent, and the observed advantages for endpoints such as heart failure are closely linked to a single research group, necessitating cautious interpretation.

**Systematic Review Registration:**

https://www.crd.york.ac.uk/PROSPERO/view/CRD420251102968, identifier CRD420251102968.

## 1 Introduction

The nondipping blood pressure (BP) pattern, characterized by a diminished nocturnal BP decline, is a recognized risk factor for major adverse cardiovascular events (MACEs) (Dong et al., 2020). This pathophysiological connection has triggered interest in the chronotherapeutic approach of administering antihypertensive medications at bedtime, with the goal of restoring the normal circadian BP rhythm and mitigating risk (Ruben et al., 2019). Early trials (Hermida et al., 2020; Hermida et al., 2010), predominantly conducted by a single research group in Spain, reported notable reductions in MACEs. However, these promising findings have not been consistently replicated in subsequent large-scale, independent studies, such as the “Treatment In the Morning *versus* Evening” (TIME) (Mackenzie et al., 2022) and “Bedtime Medication” (BedMed) trials (Garrison et al., 2025a), leading to considerable controversy and clinical uncertainty. Considering the profound implications—that a straightforward, cost-neutral adjustment in dosing time potentially yields substantial cardiovascular benefits—a comprehensive and updated synthesis of all available evidence is critical to resolve this discrepancy and inform clinical practice.

Several meta-analyses have attempted to consolidate the existing evidence on this topic. For instance, studies by Maqsood et al. (2023) and Abuel et al. (2023) offered early insights; nevertheless, their conclusions were drawn prior to the completion and publication of several recent, large-scale randomized controlled trials (RCTs), including the BedMed and BedMed-Frail trials (Garrison et al., 2025a; Garrison et al., 2025b). Incorporating these latest findings is essential to ensure that the evidence base remains current and comprehensive, providing the most up-to-date synthesis to guide clinical decision-making, even if the overall direction of effect remains unchanged.

Furthermore, existing syntheses display significant methodological variations that leave certain questions unresolved. Some analyses, such as those by Lee et al. (2024) and Wu et al. (2024), were broad in scope, encompassing a sizable number of studies (107 and 27, respectively). Nonetheless, they did not concentrate exclusively on hard cardiovascular endpoints, which may obscure the clarity of the clinical message. In contrast, other rigorous efforts, such as the meta-analysis conducted by Turgeon et al. (2025), applied strict criteria, resulting in a highly focused selection of only five large trials. Although this approach enhances internal validity, the limited number of included studies inherently restricts the capacity to thoroughly investigate sources of heterogeneity, perform meaningful subgroup analyses, or robustly assess the impact of specific study designs or patient populations. This scenario highlights the need for a synthesis that balances methodological rigor with sufficient analytical breadth.

To address the foregoing gaps in the current evidence synthesis, we conducted an updated and methodologically refined systematic review and meta-analysis. Ultimately, we aimed to (1) integrate findings from all recent major RCTs, including BedMed, into the existing body of evidence; (2) maintain a stringent focus on clinically relevant hard cardiovascular endpoints; (3) employ a comprehensive analytic approach that facilitates the exploration of heterogeneity and subgroup effects, thereby providing a nuanced and contemporary assessment of the efficacy of bedtime antihypertensive dosing.

## 2 Methods

The protocol for this study was registered with PROSPERO (CRD420251102968). The research reported in this paper adhered to the Preferred Reporting Items for Systematic Reviews and Meta-analyses (PRISMA) 2020 update (Page et al., 2021).

### 2.1 Data sources and searches

Literature searches were conducted across three databases: PubMed, EMBASE, and Web of Science. The publication time frame spanned from database inception to 16 July 2025. We utilized the following subject terms and keywords in our search: “bedtime,” “hypertension,” “death,” “cardiovascular events,” and “MACE,” among others. Detailed search strategies are provided in Supplementary Table S1 in the Supplementary Material.

### 2.2 Eligibility criteria, study selection, and data extraction

Studies were deemed eligible for inclusion if they satisfied the following criteria: (1) published as a full-length article; (2) written in English; (3) classified as an RCT; (4) included comparisons between daytime and bedtime dosing of antihypertensive medications; (5) reported risk ratios (RRs), adjusted hazard ratios (HRs), or raw data on case numbers for all-cause mortality. Studies were eligible regardless of the class of antihypertensive drugs used. Two independent reviewers screened titles and abstracts according to the inclusion criteria, reviewed full-text articles, and determined their eligibility. Missing data were requested from the corresponding authors of included articles. Any discrepancies regarding searches and selections were discussed in consultation with a third reviewer. The full text was retrieved for further inspection if a study potentially fulfilled the inclusion criteria.

Two reviewers independently conducted data extraction. The data included the following: (1) study-level general information; (2) baseline characteristics of the population; (3) outcomes from the original eligible sources, including MACEs, all-cause mortality, cardiovascular death, heart failure, myocardial infarction (MI), coronary revascularization, and clinical systolic blood pressure (SBP). The identification of clinical events was accepted as reported. Any discrepancies were verified and resolved by a third reviewer. Records of the studies were managed using EndNote X9 software.

### 2.3 Quality assessment

The methodological quality of RCTs was assessed with the “Revised Cochrane Risk of Bias Tool for Randomized Trials” (RoB-2). The certainty of evidence for the primary outcomes (MACE, cardiovascular death, and all-cause mortality) was assessed using the GRADE approach, with downgrading based on risk of bias, inconsistency, indirectness, imprecision, and publication bias.

### 2.4 Statistical analysis

Meta-analyses were performed for comparable studies using RevMan 5.4.1 software. The summary effect size for all independent events was calculated utilizing pooled RR values. For each included trial, we extracted raw event counts and total sample sizes at the protocol-defined follow-up time point for all dichotomous outcomes. These data were entered directly into RevMan software, which performed the meta-analysis using the Mantel-Haenszel method to generate pooled risk ratios (RRs) and 95% confidence intervals (CIs). No conversion from hazard ratios was required or performed. Pooled analyses were not conducted for outcomes reported in fewer than three studies. Inter-study heterogeneity was assessed using the I^2^ statistic, defined as I^2^ values ≥50%. The z statistic was computed for each outcome of interest, with results considered statistically significant at a two-sided *P*-value of <0.05. Meta-analysis results are illustrated using forest plots.

Subgroup analyses were performed to examine potential sources of heterogeneity and investigate the effects of specific variables on the results. To evaluate the robustness of the findings, we conducted a sensitivity analysis of all-cause mortality. This entailed sequentially excluding individual studies to ascertain their impact on the overall results.

Publication bias was evaluated using funnel plots to display the individual study effects for the outcomes of interest. Funnel plot asymmetry was also assessed using Egger’s test, with a two-sided *P*-value of <0.1 indicating significant publication bias. The publication bias assessments were conducted using STATA 14 software.

## 3 Results

### 3.1 Study selection and characteristics

**FIGURE 1:**
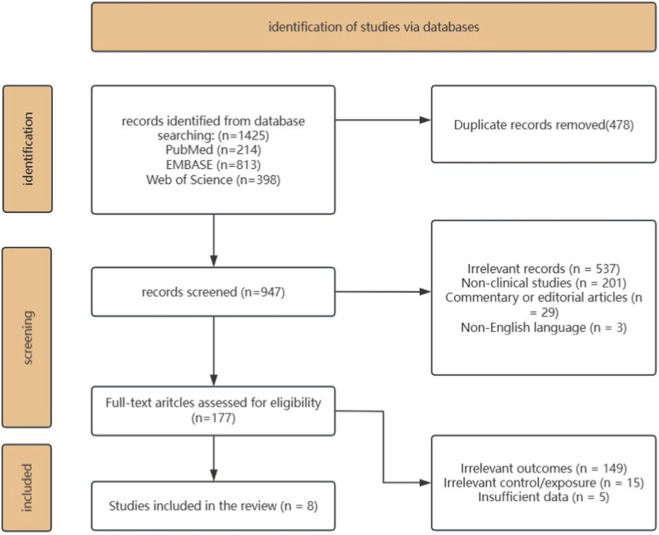
PRISMA flowchart of the screening process.

From the initial search, we identified 1,425 studies, of which 478 duplicates were discarded. Of the 947 remaining records, 770 were excluded after screening titles and abstracts owing to irrelevance to the topic or inappropriate article types. After scrutinizing the full text of the remaining 177 articles, we excluded 149 for irrelevant outcomes, 15 for irrelevant control or exposure, and 5 for reporting insufficient data. Ultimately, 8 studies satisfied the inclusion criteria and were incorporated into the systematic review (Figure 1).

On comparing bedtime and morning dosing, 64,235 patients were included. All eight included studies were randomized controlled trials (RCTs). One RCT compared bedtime amlodipine with morning fosinopril (Tatti et al., 1998), another compared bedtime verapamil with morning atenolol or hydrochlorothiazide (Black et al., 2003), while the other RCTs did not impose restrictions on the medications used. Among the studies that did not specify the drugs administered, three (Mackenzie et al., 2022; Garrison et al., 2025a; Garrison et al., 2025b) directly compared the effects of taking all antihypertensive medications at bedtime *versus* in the morning. Additionally, three RCTs (Hermida et al., 2020; Hermida et al., 2010; Ayala et al., 2013) from the same research group assessed regimens in which at least one antihypertensive medication was taken at bedtime compared with regimens where all medications were administered in the morning. All studies reported outcomes related to all-cause mortality and MACE. General information pertaining to all included studies is presented in Table 1. The average age of participants across the included studies was 63.9 years, with women constituting 48.0% of the sample. Detailed baseline characteristics are provided in Table 2.

**TABLE 1:** General characteristics of included studies.

Study ID	Country	N	Follow-up (years)	BP measurement method	Main inclusion criteria	Reported outcomes	Intervention group	Control group	Study design
Ayala et al. (2013)	Spanish	776	5.4 (median)	ABP	Resistant hypertension	①②③⑤⑥⑦⑧	>1 hypertension medication administered at bedtime	All hypertension medications ingested in the morning	Prospective, open-label, blinded-endpoint trial
Black et al. (2003) (CONVINCE)	15 countries	16,602	3 (median)	OBP	More than 55 years, hypertension plus one or more CVD risk factors	②③④⑤⑥	Bedtime verapamil with morning placebo	Morning atenolol or hydrochlorothiazide with bedtime placebo	Double-blind, randomized clinical trial conducted at multiple centers
Garrison et al. (2025a) (BedMed)	Canada	3,357	4.6 (median)	OBP	Hypertension	②④⑤	All antihypertensive medications taken at bedtime	All antihypertensive medications taken in the morning	Multicenter, open-label, pragmatic randomized clinical trial with blinded end-point assessment
Garrison et al. (2025b) (BedMed-FRAIL)	Canada	776	1.13 (median)^a^	OBP	Two or more health system encounters with hypertension	②④⑤	All antihypertensive medications taken at bedtime	All antihypertensive medications taken in the morning	Multicenter, open-label, pragmatic randomized clinical trial
Hermida et al. (2010) (MAPEC)	Spanish	2,156	5.6 (median)	ABP	Hypertension	①②③⑤⑦⑧	>1 hypertension medication administered at bedtime	Hypertension medications ingested in the morning	Prospective, open-label, blinded-endpoint trial
Hermida et al. (2020) (HYGIA)	Spanish	19,084	6.3 (median)	ABP	Hypertension	①②③④⑤⑥⑦⑧	>1 hypertension medication administered at bedtime	Hypertension medications ingested in the morning	Multicenter, controlled, PROBE study
Mackenzie et al. (2022) (TIME)	United Kingdom	21,104	5·2 (median)	OBP	Hypertension	②③④⑤	All antihypertensive medications taken at bedtime	All antihypertensive medications administered in the morning	Prospective, randomized, open-label, blinded-endpoint clinical trial
Tatti et al. (1998) (FACET)	Italy	380	2.5 (mean)	OBP	Hypertension with diabetes	①②④⑥⑦	Bedtime amlodipine	Morning fosinopril	Open-label, randomized prospective trial

^a^ Follow-up period: 415 days (1.13 years). Outcomes reported: ① All-cause mortality; ② MACE; ③ Cardiovascular death; ④ Myocardial infarction; ⑤ Heart failure; ⑥ Coronary revascularization; ⑦ Systolic blood pressure; ⑧ Non-dipper. Abbreviations: N, number of patients; ABP, ambulatory blood pressure measurement; BP, blood pressure; CVD, cardiovascular diseases; MACE, major adverse cardiovascular events; OBP, office blood pressure measurement.

**TABLE 2:** Baseline characteristics of included studies.

Study ID	Group	Number	Age (years)	Sex (female, %)	BMI	Diabetes (%)	Obstructive sleep apnea (%)	Cigarette smoking	Obesity	Chronic kidney disease (%)	Previous CVD events (%)	Clinical SBP (mmHg)	Clinical DBP (mmHg)	Awake SBP mean (mmHg)	Asleep SBP mean (mmHg)	48-h SBP mean (mmHg)	Non-dipper (%)
Ayala et al. (2013)	Morning	391	62	51.2	31.3	34.3	7.4	7.2	59.6	NA	11.3	156.0 ± 21.6	85.4 ± 11.4	131.8 ± 17.2	124.3 ± 21.2	129.3 ± 17.8	68.3
	Bedtime	385	61.2	49.1	30.7	33.2	8.1	8.6	53.0	NA	12.5	159.2 ± 23.3	87.7 ± 12.7	133.7 ± 16.6	125.6 ± 19.3	131.0 ± 16.8	63.9
Black et al. (2003) (CONVINCE)	Morning	8,361	65.6	55.8	NA	19.9	NA	22.8	49.6	NA	NA	150.1 ± 15.8	86.8 ± 9.8	NA	NA	NA	NA
	Bedtime	8,241	65.6	56.2	NA	19.7	NA	23.5	51	NA	NA	150.1 ± 16.0	86.8 ± 9.8	NA	NA	NA	NA
Garrison et al. (2025a) (BedMed)	Morning	1,680	67	56.1	28.9	18.5	20.3	7.2	NA	7.7	NA	NA	NA	NA	NA	NA	NA
	Bedtime	1,677	67	56.6	28.8	17.2	22.5	7.3	NA	7.1	NA	NA	NA	NA	NA	NA	NA
Garrison et al. (2025b) (BedMed-FRAIL)	Morning	382	88	71.5	NA	44.8	24.6	NA	NA	49	36.4	NA	NA	NA	NA	NA	NA
	Bedtime	394	88	73.4	NA	49.7	24.4	NA	NA	48.2	42.6	NA	NA	NA	NA	NA	NA
Hermida et al. (2010) (MAPEC)	Morning	1,084	56.3	50.4	30.1 ± 5.2	21.3	9.1	13.1	69.8	NA	5.3	154.4 ± 20.3	87.4 ± 11.5	134.3 ± 15.7	122.7 ± 17.4	130.7 ± 15.5	55.4
	Bedtime	1,072	55	52.7	29.8 ± 4.8	19.6	7.5	12.4	72.6	NA	5.2	155.7 ± 19.4	88.5 ± 11.0	134.2 ± 13.8	122.7 ± 15.3	130.5 ± 13.6	53
Hermida et al. (2020) (HYGIA)	Morning	9,552	60.5	43.8	29.6 ± 4.8	23.7	4.2	15.6	42.6	29.9	10.8	149.4 ± 20.5	86.3 ± 11.9	136.1 ± 14.9	123.3 ± 16.0	131.4 ± 14.4	49
	Bedtime	9,532	60.6	45	29.7 ± 4.7	24.1	3.9	14.8	43.5	28.9	10	149.5 ± 19.9	86.0 ± 12.3	135.9 ± 14.0	123.7 ± 14.6	131.7 ± 13.3	49.5
Mackenzie et al. (2022) (TIME)	Morning	10,601	65.2	42.5	NA	13.3	NA	42.6	NA	3.3	12.8	132.31 ± 11.06	77.58 ± 8.18	NA	NA	NA	NA
	Bedtime	10,503	65.0	42.5	NA	12.9	NA	41.6	NA	3.1	13.0	130.54 ± 11.02	77.15 ± 8.12	NA	NA	NA	NA
Tatti et al. (1998) (FACET)	Morning	189	62.8	36.5	30.7 ± 0.3	100	NA	13.3	NA	NA	NA	170 ± 1	95 ± 1	NA	NA	NA	NA
	Bedtime	191	63.3	44.5	30.5 ± 0.4	100	NA	19.3	NA	NA	NA	171 ± 1	94 ± 1	NA	NA	NA	NA

Abbreviations: NA, not available; CVD, cardiovascular diseases; SBP, systolic blood pressure; DBP, diastolic blood pressure; BMI, body mass index.

### 3.2 Quality assessment

**FIGURE 2:**
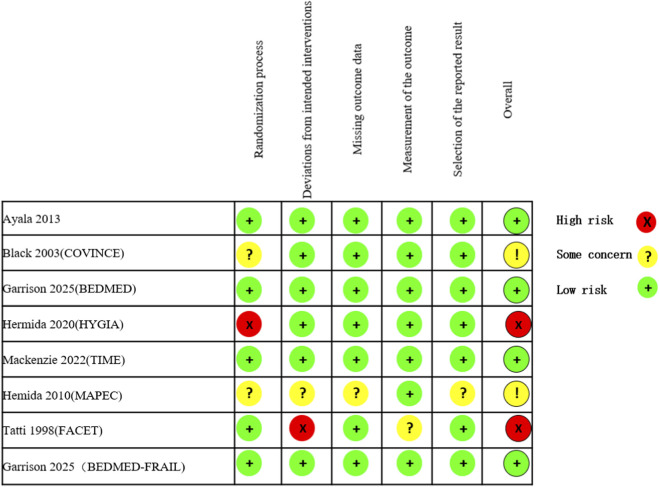
Risk of bias assessment of the included trials using Cochrane Risk of Bias Tool (ROB-2).

The RoB-2 tool was employed to evaluate the methodological quality of RCTs. Among the 8 RCTs included in the meta-analysis, four were classified as having a “low risk of bias,” two were noted to possess “some concerns,” and two were deemed to be “high risk.” Details of the quality assessment for individual studies using RoB-2 tools are illustrated in Figure 2.

The certainty of evidence for the primary outcomes was assessed using the GRADE approach. For major adverse cardiovascular events (MACE), cardiovascular death, and all-cause mortality, the evidence was rated as very low due to serious risk of bias, very serious inconsistency, and imprecision. The detailed GRADE assessment is presented in Table 3.

**TABLE 3:** GRADE evidence profile.

Certainty assessment								No of patients		Effect		Certainty	Importance
Outcomes	No of studies	Study design	Risk of bias	Inconsistency	Indirectness	Imprecision	Other considerations	Bedtime dosing	Morning dosing	Relative (95% CI)	Absolute (95% CI)		
MACE	8	Randomised trials	Very serious^a^	Very serious^b^	Not serious	Very serious^c^	Publication bias strongly suspected^d^	1913/31995 (6.0%)	2661/32240 (8.3%)	RR 0.80 (0.61–1.05)	17 fewer per 1,000 (from 32 fewer to 4 more)	⊕○○○ Very low^a,b,c,d^	CRITICAL
All cause mortality	8	Randomised trials	Very serious^a^	Very serious^e^	Not serious	Not serious	Publication bias strongly suspected^d^	1361/31995 (4.3%)	1682/32240 (5.2%)	RR 0.78 (0.59–1.04)	11 fewer per 1,000 (from 21 fewer to 2 more)	⊕○○○ Very low^a,d,e^	CRITICAL
Cardiovascular death	5	Randomised trials	Very serious^a^	Very serious^e^	Not serious	Not serious	Publication bias strongly suspected^d^	354/29733 (1.2%)	500/29989 (1.7%)	RR 0.59 (0.34–1.04)	7 fewer per 1,000 (from 11 fewer to 1 more)	⊕○○○ Very low^a,d,e^	CRITICAL

Abbreviations: CI: confidence interval; RR: risk ratio a. Serious risk of bias (especially Hermida group limitations) b. Very serious inconsistency (I^2^ = 94%; resolved in non-Hermida subgroup: I^2^ = 24%) c. Very serious imprecision (fragile finding: exclusion of any one of 3 non-Hermida trials yielded significance) d. Suspected publication bias (positive results dominated by single research group) e. Very serious inconsistency (I^2^ = 91%; resolved in non-Hermida subgroup: I^2^ = 0%).

### 3.3 Outcomes

#### 3.3.1 Primary outcomes

##### 3.3.1.1 All-cause mortality

**FIGURE 3:**
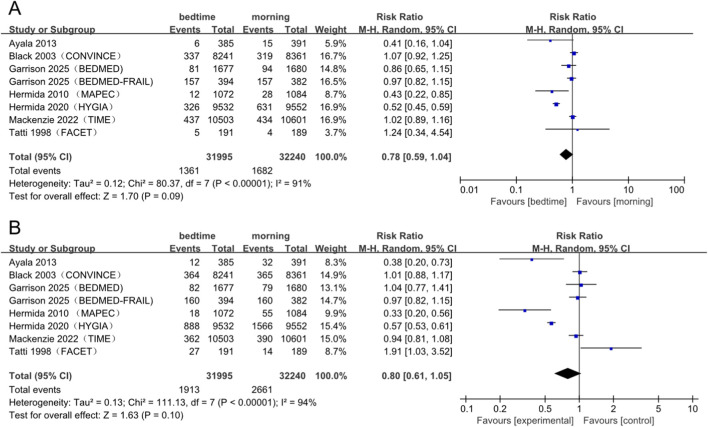
Forest plot of the primary outcomes **(A)** All-cause mortality and **(B)** MACE. MACE, major adverse cardiovascular events; CI, confidence interval; RR, risk ratio.

The overall analysis, which included 8 studies, indicated a non-significant trend toward benefit, with a pooled RR of 0.78 (95% CI: 0.59–1.04; *P* = 0.09). However, substantial heterogeneity was observed (I^2^ = 91%; Figure 3).

Subgroup analysis revealed a notable divergence in results. Studies from the Hermida trials demonstrated a significant 49% reduction in risk (RR: 0.51; 95% CI: 0.45–0.58; *P* < 0.00001) with no observed heterogeneity (I^2^ = 0%). In contrast, other trials yielded no significant effects (RR: 1.01; 95% CI: 0.93–1.09; *P* = 0.83), similarly demonstrating no heterogeneity (I^2^ = 0%; Supplementary Figure S2).

The test for subgroup differences was considerably significant (χ^2^ = 77.51, df = 1, *P* < 0.00001; I^2^ = 98.7%), suggesting that the methodology or population characteristics of the Hermida trials are major contributors to overall heterogeneity and serve as critical effect modifiers.

##### 3.3.1.2 Major adverse cardiac events

**FIGURE 4:**
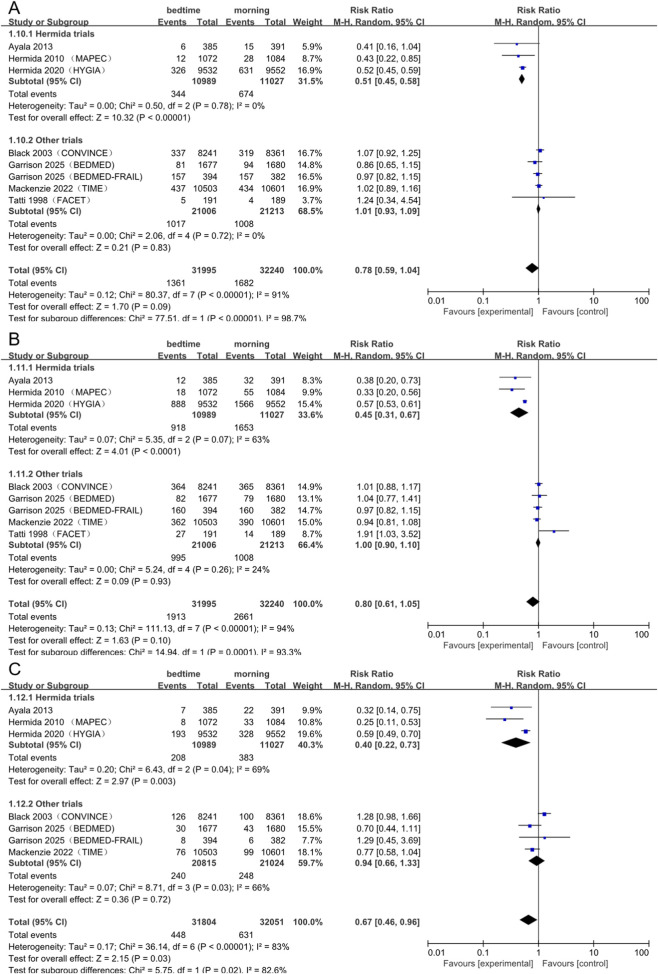
Forest plot of the subgroup analysis **(A)** All-cause mortality **(B)** MACE and **(C)** Heart failure. CI, confidence interval; RR, risk ratio.

The overall meta-analysis did not show a statistically significant reduction in risk (RR: 0.80; 95% CI: 0.61–1.05; *P* = 0.10). Similarly, substantial heterogeneity was noted (I^2^ = 94%; Figure 4). While visual inspection of the funnel plot indicated a certain degree of asymmetry (Supplementary Figure S4), Egger’s regression test did not provide statistical evidence for publication bias (*P* = 0.463; Supplementary Figure S6).

In subgroup analyses, studies conducted by the Hermida group demonstrated a notably significant benefit (RR: 0.45; 95% CI: 0.31–0.67; *P* < 0.00001; I^2^ = 63%). In contrast, studies from other groups exhibited no effect and displayed minimal heterogeneity (RR: 1.00; 95% CI: 0.90–1.10; *P* = 0.93; I^2^ = 24%). The difference observed between these subgroups exhibited a high degree of statistical significance (I^2^ for subgroup differences = 93.3%, *P* = 0.0001; Figure 4).

#### 3.3.2 Secondary outcomes

##### 3.3.2.1 Secondary cardiovascular outcomes

The pooled analysis revealed a significantly positive effect of bedtime dosing on heart failure (RR: 0.67; 95% CI: 0.46–0.96; *P* = 0.03), albeit with considerable heterogeneity (I^2^ = 83%). This overall effect was primarily influenced by a sizable reduction in risk observed exclusively in the Hermida group studies (RR: 0.40; 95% CI: 0.22–0.73; *P* = 0.003). This finding contradicted the null effect reported in other studies (RR: 0.94; 95% CI: 0.66–1.33; *P* = 0.72), highlighting a significant subgroup difference (Supplementary Figure S1).

Regarding other endpoints, analyses indicated non-significant trends favoring bedtime dosing for cardiovascular death (RR: 0.59; 95% CI: 0.34–1.04; *P* = 0.07), myocardial infarction (RR: 0.85; 95% CI: 0.70–1.03; *P* = 0.10), and coronary revascularization (RR: 0.79; 95% CI: 0.50–1.24; *P* = 0.30; Figure 4).

##### 3.3.2.2 Blood pressure

A substantial decline in office SBP was observed with bedtime dosing relative to morning dosing (mean difference: −3.27 mmHg; 95% CI: −4.13 to −2.41; *P* < 0.00001). Additionally, non-dipper status was solely reported by the Hermida group, which discovered that bedtime dosing significantly alleviated the risk (RR: 0.61; 95% CI: 0.47–0.77; *P* < 0.0001; Supplementary Figure S1).

### 3.4 Sensitivity analysis

Sensitivity analysis for all-cause mortality remained robust, with no substantial changes in effect size or statistical significance upon sequential exclusion of individual studies. In contrast, the significant benefit observed for heart failure was entirely dependent on the inclusion of studies from the Hermida group; exclusion of any one of these trials rendered the result non-significant. Conversely, for MACE, cardiovascular death, and myocardial infarction, the pooled estimates were sensitive to the exclusion of specific non-Hermida trials (Tatti, CONVINCE, and BedMed, respectively), with significance emerging accordingly. The analysis of office blood pressure was not materially altered in any of the sensitivity analyses performed (Supplementary Figure S5).

## 4 Discussion

This meta-analysis of recent randomized and prospective studies established no significant reduction in the rates of all-cause mortality, MACE, cardiovascular death, myocardial infarction, or coronary revascularization associated with the bedtime administration of antihypertensive medication. Although statistically significant reductions were observed for the composite endpoint of heart failure, the finding was accompanied by overwhelming heterogeneity (I^2^ = 83%). To decipher the origin of this heterogeneity, we conducted a prespecified subgroup analysis that stratified trials according to their originating research group. This analysis revealed a clear pattern: the heterogeneity was predominantly attributable to studies conducted by the Hermida research group. On analyzing these studies separately, the heterogeneity within the Hermida and non-Hermida subgroups markedly decreased to low or moderate levels (for example, the I^2^ statistic for MACE decreased from 94% to 24% in the non-Hermida subgroup). Moreover, the significant benefit for heart failure was entirely driven by the inclusion of trials from the Hermida group; the statistical significance disappeared when these key early studies were removed, underscoring the fragility of this finding and its dependence on a single investigative team.

The instability of the pooled estimates is further illustrated by a reciprocal sensitivity pattern. The significant benefit observed for heart failure was contingent on the inclusion of studies from the Hermida group, whereas the non-significant findings for MACE, cardiovascular death, and myocardial infarction became significant upon exclusion of specific non-Hermida trials. This bidirectional sensitivity, where the statistical significance of key outcomes depends entirely on the inclusion or exclusion of one research subgroup, underscores a considerable lack of consensus, indicating the fragility and internal inconsistency of the overall evidence base. Therefore, the apparent overall benefit for MACE and heart failure may be predominantly influenced by results from this single investigative team. Considering the well-documented methodological concerns regarding various aspects of their trial conduct—including patient selection, randomization, and endpoint adjudication—the promising effects reported in these early trials are potentially undermined by limitations related to internal validity. Consequently, the current body of evidence does not robustly corroborate a broad cardioprotective benefit of bedtime antihypertensive dosing across patient populations.

Several independent studies have raised methodological concerns regarding the reports from Hermida et al. (Kreutz et al., 2020; Brunström et al., 2021; Lüscher et al., 2020; Ladebo et al., 2024). Critical examinations of baseline characteristics have revealed imbalances in numerous fundamental parameters between the morning and bedtime dosing groups (Kreutz et al., 2020), such as BMI, diastolic blood pressure, age, and waist circumference. Furthermore, deviations from the prespecified protocol were observed in certain instances (Brunström et al., 2021). For instance, in the HYGIA trial, the study population increased from 5,000 to over 19,000 participants without explanation—despite a statistical power calculation indicating that 10,000 patients would be sufficient. Moreover, the trials reported consistently high participant retention, with attrition rates as low as 0%–3.5% reported over the follow-up period. The researchers evidently did not employ an independent data monitoring committee for the ongoing monitoring of outcomes (Lüscher et al., 2020). Collectively, these factors raise concerns regarding potential unaddressed sources of bias, including the possibility of undeclared conflicts of interest that might have influenced the conduct or reporting of the study (Lüscher et al., 2020).

A fundamental methodological feature of our analysis is its exclusive focus on hard cardiovascular endpoints. Consequently, we excluded studies solely reporting ambulatory blood pressure monitoring (ABPM) parameters without clinical outcome data. Although the impact of bedtime dosing on ABPM metrics, such as nocturnal dipping, has been extensively investigated in other syntheses (Wu et al., 2024), we opted to limit our examination of blood pressure to office SBP and the prevalence of non-dipper patterns to ensure analytical consistency and clinical relevance. Nonetheless, this focused approach has its limitations: it precludes a detailed mechanistic exploration of whether modifications in circadian BP profiles mediate any potential treatment effects. A growing body of evidence suggests that the non-dipper BP pattern and morning hypertension are robustly linked to adverse outcomes (Kario et al., 2014; Staplin et al., 2023). Importantly, the improvement of a surrogate marker does not necessarily guarantee a proportional reduction in cardiovascular risk—a principle underlined by large outcome studies such as the BedMed trial. In our meta-analysis, data on the restoration of a normal circadian BP profile were predominantly reported by the Hermida group. Other major trials did not stratify outcomes according to the baseline BP patterns of participants (for example, dipper *versus* non-dipper *versus* extreme dipper). As a result, we could not determine whether the differential treatment effects observed—particularly the benefits reported by a single research group—are attributed to a selective correction of abnormal circadian rhythms in a responsive subpopulation. This remains a critical question for future research.

Despite theoretical concerns that bedtime dosing may lead to nocturnal hypotension (Melgarejo et al., 2018), potentially posing risks of cerebral hypoperfusion (associated with falls, fractures, and cognitive decline) and diminished ocular perfusion pressure (a factor in glaucoma), evidence from large RCTs does not directly support these concerns. TIME and BedMed trials included in our analysis did not find a significant increase in the rates of these specific events between dosing groups, indicating that the undesirable clinical sequelae of nocturnal hypoperfusion did not manifest. Beyond safety, practical implementation considerations are also important. In the TIME trial, approximately 30% of participants did not consistently adhere to their assigned dosing time, with non-adherence being significantly more frequent in the bedtime group than in the morning group (39% *versus* 22.5%; *P* < 0.0001).

The present analysis incorporated studies with a potential source of methodological heterogeneity: comparisons involving different antihypertensive agents. This variability in pharmacotherapy was inherent to our research question, which aimed to evaluate the dosing-time strategy itself rather than the efficacy of specific drugs. Regarding pharmacological differences, authoritative evidence supports the comparability of certain contrasts; for instance, the INVEST trial (Cooper-DeHoff et al., 2009) established the cardiovascular outcome equivalence of atenolol- and verapamil-based strategies, which underpins the analysis of the CONVINCE trial. For other drug comparisons lacking direct equivalence data, their influence was tested in sensitivity analyses.

Our analysis encompassed diverse patient populations, including those with diabetes, resistant hypertension, and frail older adults. Moreover, existing literature suggests that hypertension outcomes can be influenced by various comorbidities, such as glaucoma (Pillunat et al., 2015), asymptomatic cerebral infarction/cognitive decline (Vermeer et al., 2007), silent myocardial ischemia (Pierdomenico et al., 1998), obstructive sleep apnea (Kasiakogias et al., 2015), isolated nocturnal hypertension (Tadic et al., 2020), and hypertension-mediated organ damage (Salles et al., 2016; Kario et al., 2020; Yan et al., 2015). However, the current body of evidence is inadequate to draw definitive conclusions regarding these specific subgroups. In the present study, we could not conduct further stratified analyses owing to limitations in data availability, inconsistent reporting of comorbidities across primary studies, and concerns regarding statistical power.

Overall, although bedtime and morning dosing exhibit comparable efficacy, the World Hypertension League does not endorse the routine adoption of bedtime dosing (Stergiou et al., 2022). Considering the methodological concerns, risk of bias, and conflicting findings between studies conducted by Hermida et al. and others, there is currently insufficient justification for adopting their recommendations. A pragmatic approach would be to select the dosing time that is most convenient for the patient, thereby enhancing adherence.

## 5 Conclusion

This updated meta-analysis, focusing on hard cardiovascular endpoints, reveals that the existing evidence does not convincingly support a clinically meaningful advantage of routinely transitioning antihypertensive medication to bedtime. While a statistically significant reduction was observed for the composite outcome of heart failure, this finding was marked by considerable heterogeneity and was heavily reliant on studies from a single research group, rendering it fragile. No significant advantages were observed for all-cause mortality, MACE, cardiovascular death or myocardial infarction. Notably, large RCTs, such as TIME and BedMed, reported no increased risk of harm (for example, falls and fractures) associated with bedtime dosing. The fundamental inconsistency in the evidence base—where apparent benefits are neither replicable across independent research groups nor stable in sensitivity analyses—suggests that the observed signal is likely attributable to methodological factors and bias rather than a genuine biological effect of dosing time. Consequently, while bedtime dosing is deemed safe, its broader cardiovascular efficacy remains uncertain and may depend on unverified specific practices. Future studies should standardize and report ABPM outcomes to substantiate this potential and elucidate the underlying mechanisms.

## 6 Limitations

The interpretation of our findings is influenced by several limitations. The trials included varied in methodological quality, with a cluster of studies from a single group disproportionately contributing to the positive signal while demonstrating a higher risk of bias. Significant statistical and clinical heterogeneity persists, partially owing to variations in antihypertensive regimens across the trials. Finally, the analysis is limited by the use of aggregate data, precluding a patient-level exploration of major effect modifiers such as baseline circadian blood pressure patterns.

## Data Availability

The original contributions presented in the study are included in the article/Supplementary Material, further inquiries can be directed to the corresponding authors.
