# Supplementary material for: Timing of antihypertensive medication (bedtime versus morning) and cardiovascular risk: an updated systematic review and meta-analysis
Source: Front Pharmacol. 2026 Mar 30;17:1758890. doi: 10.3389/fphar.2026.1758890 (PMC13070909; doi:10.3389/fphar.2026.1758890)
Supplement: Supplementary file 1 [file DataSheet2.pdf]

FigS1 Forest plot of the secondary outcomes
FigS2 Forest plot of the subgroup analysis
FigS3 Funnel Plot of primary outcome
FigS4 Funnel Plot of secondary outcome
FigS5 Leave-one-out sensitivity analysis
FigS6 Publication Bias analysis STATA Analysis

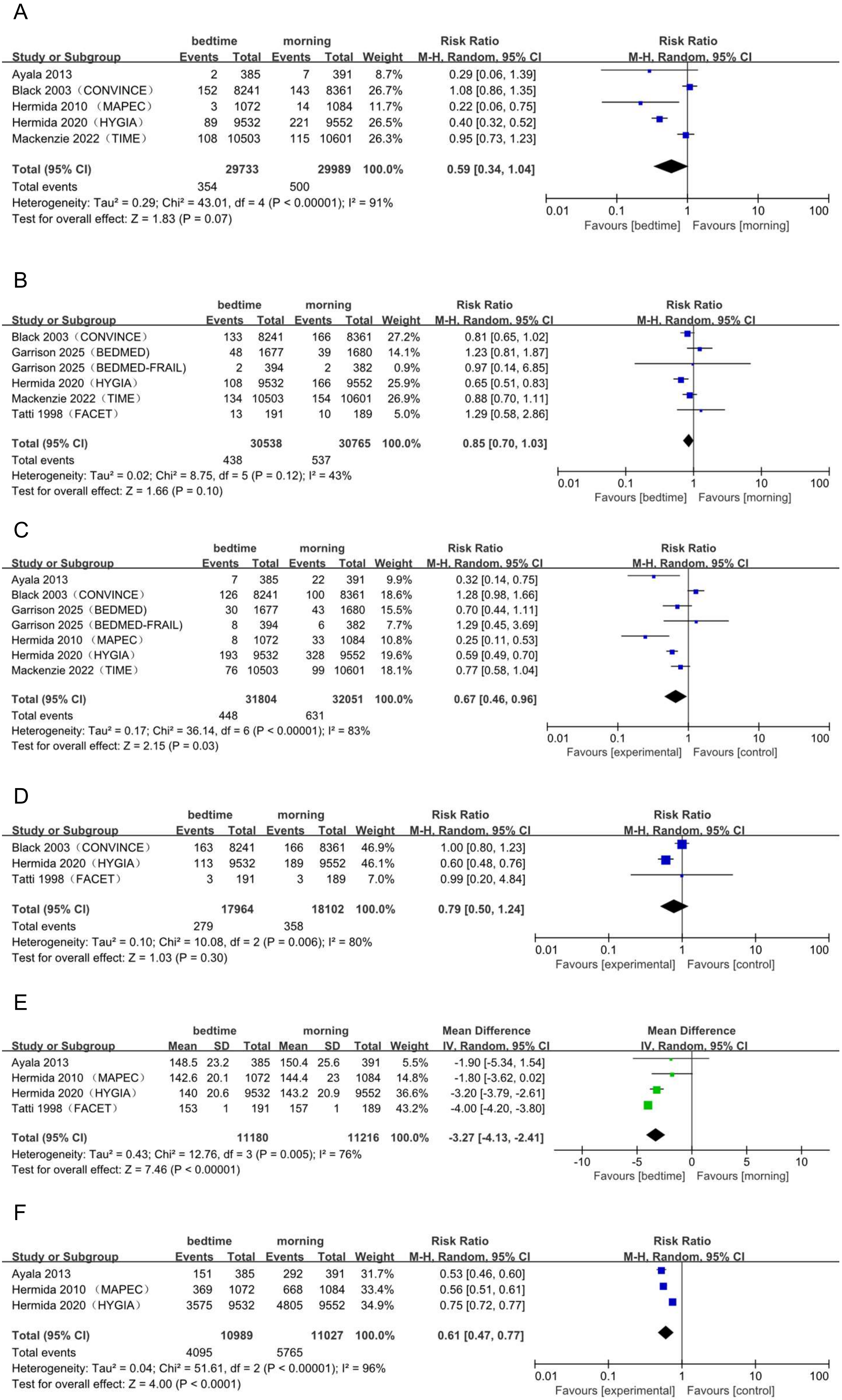
A
bedtime
morning
Risk Ratio
Risk Ratio
Study or Subgroup Events Total Events Total Weight M-H, Random, 95% CI M-H, Random, 95% CI
Ayala 2013 2 385 7 391 8.7% 0.29 [0.06, 1.39]
Black 2003（CONVINCE） 152 8241 143 8361 26.7% 1.08 [0.86, 1.35]
Hermida 2010 （MAPEC） 3 1072 14 1084 11.7% 0.22 [0.06, 0.75]
Hermida 2020（HYGIA） 89 9532 221 9552 26.5% 0.40 [0.32, 0.52]
Mackenzie 2022（TIME） 108 10503 115 10601 26.3% 0.95 [0.73, 1.23]
Total (95% CI) 29733 29989 100.0% 0.59 [0.34, 1.04]
Total events 354 500
Heterogeneity: Tau² = 0.29; Chi² = 43.01, df = 4 (P < 0.00001); I² = 91%
Test for overall effect: Z = 1.83 (P = 0.07)
0.01 0.1 1 10 100
Favours [bedtime] Favours [morning]
B
bedtime
morning
Risk Ratio
Risk Ratio
Study or Subgroup Events Total Events Total Weight M-H, Random, 95% CI M-H, Random, 95% CI
Black 2003（CONVINCE） 133 8241 166 8361 27.2% 0.81 [0.65, 1.02]
Garrison 2025（BEDMED) 48 1677 39 1680 14.1% 1.23 [0.81, 1.87]
Garrison 2025（BEDMED-FRAIL) 2 394 2 382 0.9% 0.97 [0.14, 6.85]
Hermida 2020（HYGIA） 108 9532 166 9552 25.9% 0.65 [0.51, 0.83]
Mackenzie 2022（TIME） 134 10503 154 10601 26.9% 0.88 [0.70, 1.11]
Tatti 1998（FACET） 13 191 10 189 5.0% 1.29 [0.58, 2.86]
Total (95% CI) 30538 30765 100.0% 0.85 [0.70, 1.03]
Total events 438 537
Heterogeneity: Tau² = 0.02; Chi² = 8.75, df = 5 (P = 0.12); I² = 43%
Test for overall effect: Z = 1.66 (P = 0.10)
0.01 0.1 1 10 100
Favours [bedtime] Favours [morning]
C
bedtime
morning
Risk Ratio
Risk Ratio
Study or Subgroup Events Total Events Total Weight M-H, Random, 95% CI M-H, Random, 95% CI
Ayala 2013 7 385 22 391 9.9% 0.32 [0.14, 0.75]
Black 2003（CONVINCE） 126 8241 100 8361 18.6% 1.28 [0.98, 1.66]
Garrison 2025（BEDMED) 30 1677 43 1680 15.5% 0.70 [0.44, 1.11]
Garrison 2025（BEDMED-FRAIL) 8 394 6 382 7.7% 1.29 [0.45, 3.69]
Hermida 2010 （MAPEC） 8 1072 33 1084 10.8% 0.25 [0.11, 0.53]
Hermida 2020（HYGIA） 193 9532 328 9552 19.6% 0.59 [0.49, 0.70]
Mackenzie 2022（TIME） 76 10503 99 10601 18.1% 0.77 [0.58, 1.04]
Total (95% CI) 31804 32051 100.0% 0.67 [0.46, 0.96]
Total events 448 631
Heterogeneity: Tau² = 0.17; Chi² = 36.14, df = 6 (P < 0.00001); I² = 83%
Test for overall effect: Z = 2.15 (P = 0.03)
0.01 0.1 1 10 100
Favours [experimental] Favours [control]
D
bedtime
morning
Risk Ratio
Risk Ratio
Study or Subgroup Events Total Events Total Weight M-H, Random, 95% CI M-H, Random, 95% CI
Black 2003（CONVINCE） 163 8241 166 8361 46.9% 1.00 [0.80, 1.23]
Hermida 2020（HYGIA） 113 9532 189 9552 46.1% 0.60 [0.48, 0.76]
Tatti 1998（FACET） 3 191 3 189 7.0% 0.99 [0.20, 4.84]
Total (95% CI) 17964 18102 100.0% 0.79 [0.50, 1.24]
Total events 279 358
Heterogeneity: Tau² = 0.10; Chi² = 10.08, df = 2 (P = 0.006); I² = 80%
Test for overall effect: Z = 1.03 (P = 0.30)
0.01 0.1 1 10 100
Favours [experimental] Favours [control]
E
bedtime
morning
Mean Difference
Mean Difference
Study or Subgroup Mean SD Total Mean SD Total Weight IV, Random, 95% CI IV, Random, 95% CI
Ayala 2013 148.5 23.2 385 150.4 25.6 391 5.5% -1.90 [-5.34, 1.54]
Hermida 2010 （MAPEC） 142.6 20.1 1072 144.4 23 1084 14.8% -1.80 [-3.62, 0.02]
Hermida 2020（HYGIA） 140 20.6 9532 143.2 20.9 9552 36.6% -3.20 [-3.79, -2.61]
Tatti 1998（FACET） 153 1 191 157 1 189 43.2% -4.00 [-4.20, -3.80]
Total (95% CI) 11180 11216 100.0% -3.27 [-4.13, -2.41]
Heterogeneity: Tau² = 0.43; Chi² = 12.76, df = 3 (P = 0.005); I² = 76%
Test for overall effect: Z = 7.46 (P < 0.00001)
-10 -5 0 5 10
Favours [bedtime] Favours [morning]
F
bedtime
morning
Risk Ratio
Risk Ratio
Study or Subgroup Events Total Events Total Weight M-H, Random, 95% CI M-H, Random, 95% CI
Ayala 2013 151 385 292 391 31.7% 0.53 [0.46, 0.60]
Hermida 2010 （MAPEC） 369 1072 668 1084 33.4% 0.56 [0.51, 0.61]
Hermida 2020（HYGIA） 3575 9532 4805 9552 34.9% 0.75 [0.72, 0.77]
Total (95% CI) 10989 11027 100.0% 0.61 [0.47, 0.77]
Total events 4095 5765
Heterogeneity: Tau² = 0.04; Chi² = 51.61, df = 2 (P < 0.00001); I² = 96%
Test for overall effect: Z = 4.00 (P < 0.0001)
0.01 0.1 1 10 100
Favours [experimental] Favours [control]

Figure S1. Forest plot of the secondary outcomes (a cardiovascular death, b MI, c heart failure, d coronary revascularization, e SBP, f non-dipper.) MI, myocardial infarction; CI, confidence interval; RR, risk ratio.

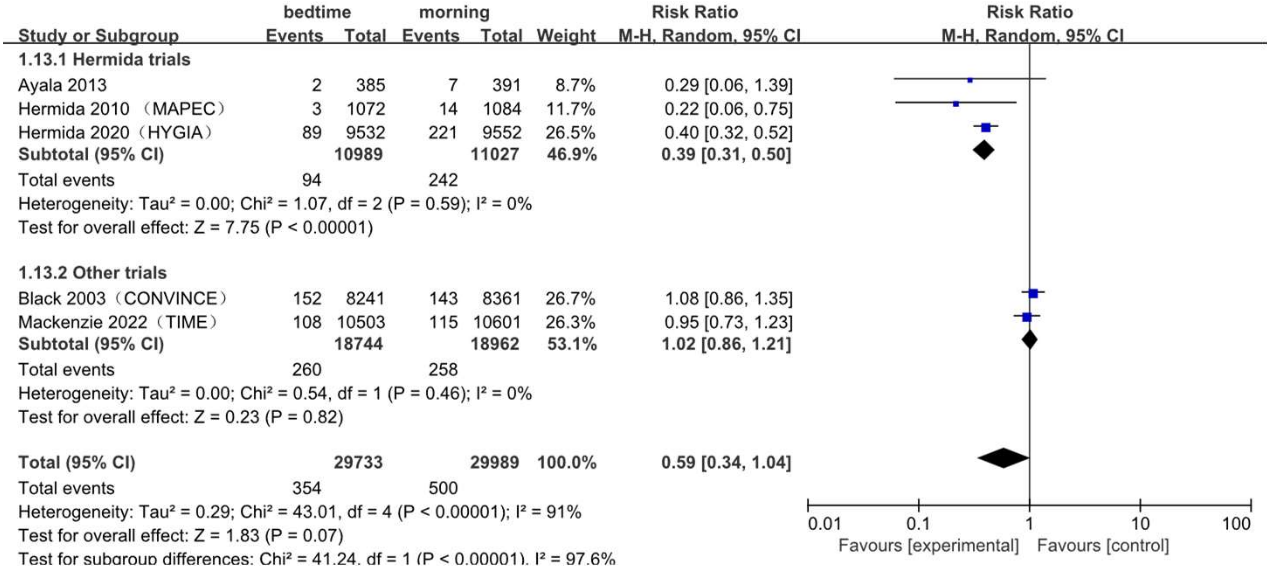

Figure S2 Forest plot of the subgroup analysis of cardiovascular death. CI, confidence interval; RR, risk ratio.

A

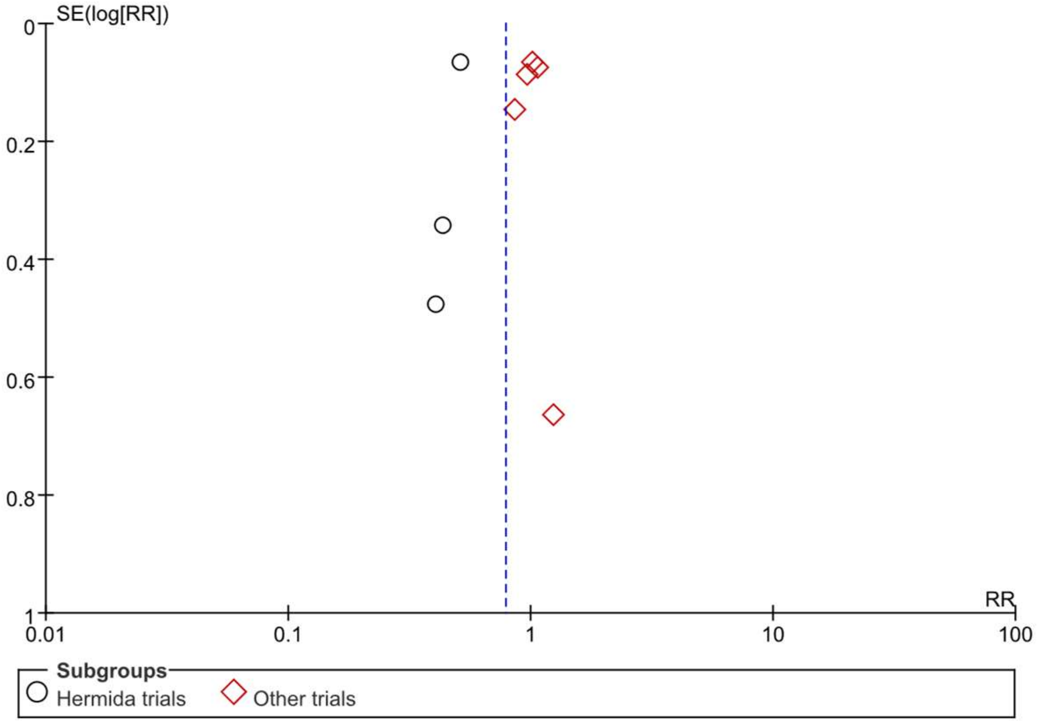

B

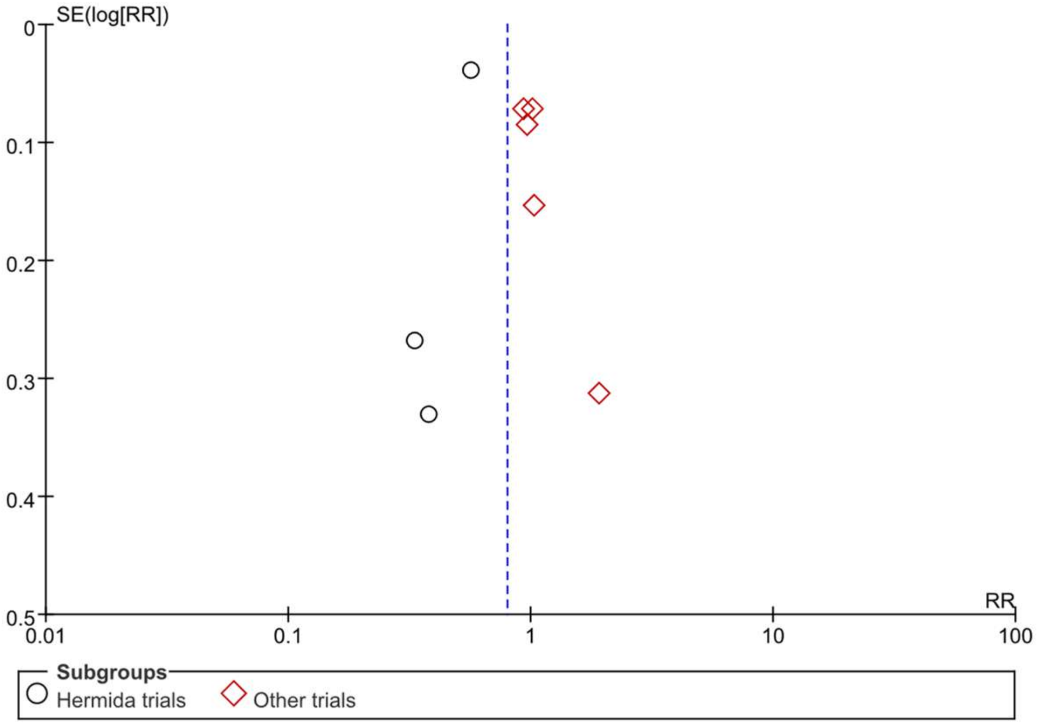

Figure S3. Funnel Plot of primary outcome (A all-cause mortality, B MACE.)

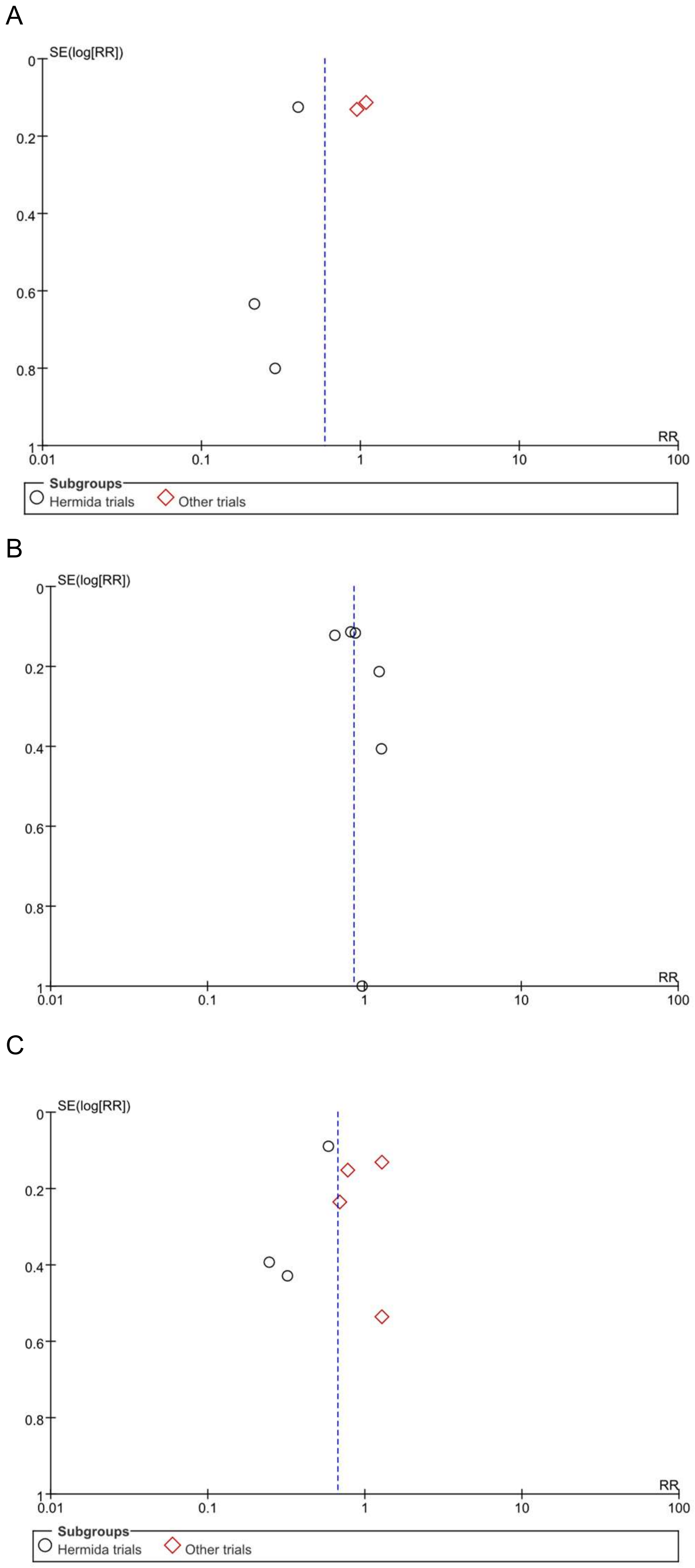

Figure S4. Funnel Plot of secondary outcome (A cardiovascular death, B MI, C heart failure.)

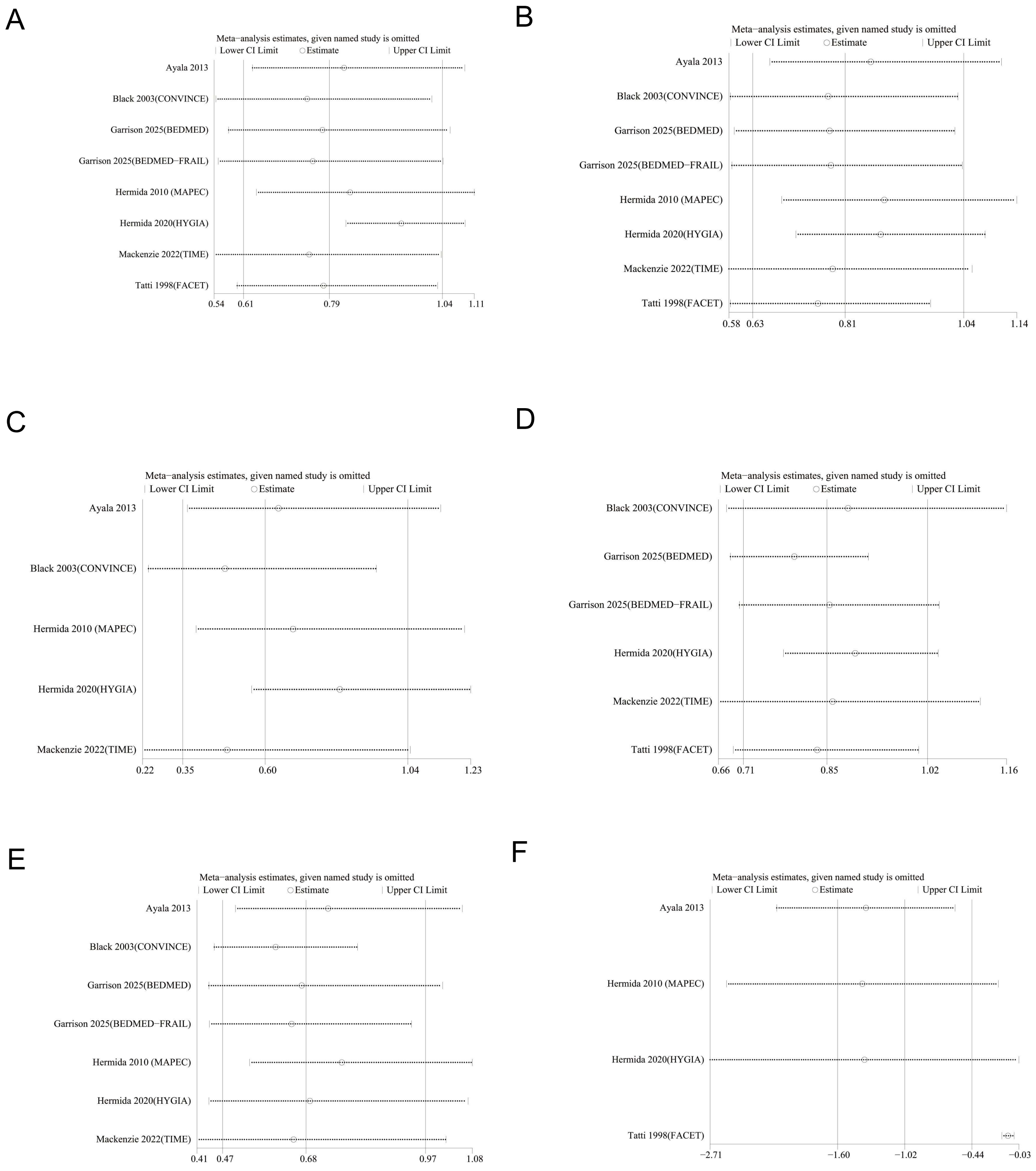

Figure S5. Leave-one-out sensitivity analysis (A all-cause mortality, B MACE, C cardiovascular death, D MI, E heart failure, F SBP.)

A

```
Egger's test for small-study effects:
Regress standard normal deviate of intervention
  effect estimate against its standard error

.
Number of studies =  8                                 Root MSE      =   3.655
```

Std_Eff	Coefficient	Std. err.	t	P>|t|	[95% conf.	interval]
slope	-.4028048	.181123	-2.22	0.068	-.8459968	.0403872
bias	1.742119	2.22416	0.78	0.463	-3.700204	7.184442

```
Test of H0: no small-study effects          P = 0.463
```

B

```
Egger's test for small-study effects:
Regress standard normal deviate of intervention
  effect estimate against its standard error

.
Number of studies =  8                                 Root MSE      =   3.473
```

Std_Eff	Coefficient	Std. err.	t	P>|t|	[95% conf.	interval]
slope	-.1422861	.2271572	-0.63	0.554	-.6981198	.4135476
bias	-.4865573	2.261449	-0.22	0.837	-6.020124	5.047009

```
Test of H0: no small-study effects          P = 0.837
```

Figure S6. STATA Publication Bias （A all-cause mortality, B MACE.)
