# Supplementary material for: Timing of antihypertensive medication (bedtime versus morning) and cardiovascular risk: an updated systematic review and meta-analysis
Source: Front Pharmacol. 2026 Mar 30;17:1758890. doi: 10.3389/fphar.2026.1758890 (PMC13070909; doi:10.3389/fphar.2026.1758890)
Supplement: Supplementary file 2 [file DataSheet1.pdf]

Search strategy.

Database	Search strategy
PubMed (n=214)	(("bedtime dosing"[tiab] OR "evening dosing"[tiab] OR "nighttime dosing"[tiab] OR "chronotherapy"[tiab] OR "dosing time"[tiab] OR "timing of medication"[tiab] OR "morning dosing"[tiab] OR "awakening dosing"[tiab] OR "daytime dosing"[tiab]) AND ("hypertension"[tiab] OR "high blood pressure"[tiab] OR "antihypertensive"[tiab] OR "blood pressure"[tiab]) AND ("MACE"[tiab] OR "Major adverse cardiovascular events"[tiab] OR "cardiovascular events"[tiab] OR "MI"[tiab] OR "myocardial infarction"[tiab] OR "Mortality"[tiab] OR "Death"[tiab] OR "cardiovascular death"[tiab] OR "Blood pressure"[tiab] OR "systolic pressure"[tiab] OR "diastolic pressure"[tiab]) AND "Journal Article"[pt]) NOT ("Preprint"[pt] OR ("Review"[pt] OR "Case Reports"[pt] OR "Editorial"[pt] OR "News"[pt] OR "Published Erratum"[pt] OR "Comment"[pt] OR "Letter"[pt]))
EMBASE (n=813)	('bedtime dosing':ti,ab OR 'evening dosing':ti,ab OR 'nighttime dosing':ti,ab OR 'chronotherapy':ti,ab OR 'dosing time':ti,ab OR 'timing of medication':ti,ab OR 'morning dosing':ti,ab OR 'awakening dosing':ti,ab OR 'daytime dosing':ti,ab OR 'chronotherapy'/exp OR 'chronotherapy') AND ('hypertension':ti,ab OR 'high blood pressure':ti,ab OR 'antihypertensive':ti,ab OR 'blood pressure':ti,ab OR 'essential hypertension'/exp OR 'essential hypertension' OR 'hypertension'/exp OR 'hypertension' OR 'antihypertensive agent'/exp OR 'antihypertensive agent' OR 'blood pressure'/exp OR 'blood pressure') AND ('mace':ti,ab OR 'major adverse cardiovascular events':ti,ab OR 'cardiovascular events':ti,ab OR 'mi':ti,ab OR 'myocardial infarction':ti,ab OR 'mortality':ti,ab OR 'death':ti,ab OR 'cardiovascular death':ti,ab OR 'blood pressure':ti,ab OR 'systolic pressure':ti,ab OR 'diastolic pressure':ti,ab OR 'major adverse cardiovascular event'/exp OR 'major adverse cardiovascular event' OR 'cardiovascular disease'/exp OR 'cardiovascular disease' OR 'myocardial infarction'/exp OR 'myocardial infarction' OR 'mortality'/exp OR 'mortality' OR 'cause of death'/exp OR 'cause of death' OR 'cardiovascular mortality'/exp OR 'cardiovascular mortality' OR 'blood pressure'/exp OR 'blood pressure' OR 'systolic blood pressure'/exp OR 'systolic blood pressure' OR 'diastolic blood pressure'/exp OR 'diastolic blood pressure') NOT ('review'/exp OR 'review' OR 'case report'/exp OR 'case report' OR 'editorial'/exp OR 'editorial' OR 'news'/exp OR 'news' OR 'erratum'/exp OR 'erratum' OR 'comment' OR 'letter'/exp OR 'letter')
Web of Science (n=398)	(((TS=("bedtime dosing" OR "evening dosing" OR "nighttime dosing" OR "chronotherapy" OR "dosing time" OR "timing of medication" OR "morning dosing" OR "awakening dosing" OR "daytime dosing"))<br>AND(TS=("hypertension" OR "high blood pressure" OR "antihypertensive" OR "blood pressure"))<br>AND(TS=("MACE" OR "Major adverse cardiovascular events" OR "cardiovascular events" OR "MI" OR "myocardial infarction" OR "Mortality" OR "Death" OR "cardiovascular death" OR "Blood pressure" OR "systolic pressure" OR "diastolic pressure")))<br>AND DT=Article

NOT DT=(Review OR "Case Report" OR Editorial OR "News Item" OR Correction OR "Letter" OR "Book Chapter")) NOT (SILOID==("PPRN"))
